# Outcomes and challenges in the surgical treatment of trans-olecranon fracture-dislocations: A case series study

**DOI:** 10.1016/j.ijscr.2025.111254

**Published:** 2025-04-05

**Authors:** Edgar Barros, Eduardo Noboa, Carlos Peñaherrera, Francisco Endara, Paul Vaca Perez, Diego Michilena, Alejandro Barros Castro

**Affiliations:** a Department of Orthopedics and Traumatology, Hospital Voz Andes, Quito, Ecuador; b Department of Orthopedics and Traumatology, Hospital Metropolitano, Quito, Ecuador; c International University of Ecuador in the Metropolitan Hospital, Quito, Ecuador

**Keywords:** Elbow joint, Fracture fixation, Elbow dislocation, Patient-reported outcome measures (PROMS)

## Abstract

**Introduction:**

Transolecranon fracture-dislocations represent a complex subset of elbow injuries characterized by concomitant fractures of the olecranon, coronoid process, and radial head, often associated with posterior dislocation. These injuries pose significant surgical challenges due to their inherent instability and high risk of poor functional outcomes. Despite previous studies describing different fixation techniques, there is no standardized surgical protocol to optimize outcomes. This study aims to evaluate the clinical and functional results of a structured surgical approach in the management of these injuries.

**Methods:**

A retrospective case series was conducted, analyzing 12 patients with transolecranon fracture-dislocations treated at two orthopedic centers. Patients underwent open reduction and internal fixation (ORIF) using a standardized fixation sequence through the Wrightington posterolateral approach, with selective use of the anteromedial approach when necessary. Functional outcomes were assessed using the Mayo Elbow Performance Score (MEPS), Disabilities of the Arm, Shoulder, and Hand (DASH) score, and the Oxford Elbow Score (OES) at 2, 6, 12, and 24 months postoperatively. Radiographic follow-up included evaluation of bone healing, joint congruence, and post-traumatic arthritis. Statistical analysis was performed using repeated-measures ANOVA to assess functional improvement over time.

**Results:**

At a mean follow-up of 24 months, significant improvement in functional scores was observed. MEPS increased from 63 at two months to 90 at 24 months, while DASH scores decreased from 45 to 15, and OES improved from 28 to 46 over the same period. Range of motion showed progressive recovery, with mean flexion-extension reaching 160° of flexion and full extension at final follow-up. All fractures achieved radiographic consolidation, with no cases of implant failure. Despite evident clinical improvement, statistical significance was not achieved in ANOVA analysis (*p* > 0.05), likely due to sample size limitations.

**Conclusion:**

A standardized surgical approach combining structured fixation strategies and early rehabilitation provides favorable clinical and functional outcomes in transolecranon fracture-dislocations. The results of this study reinforce the importance of anatomical reduction and stable fixation in restoring elbow function. Future studies with larger sample sizes are needed to further validate these findings and refine treatment protocols.

**Level of evidence:**

IV

## Highlights

• **A standardized surgical approach** involving sequential fixation and selective dual-approach exposure leads to improved clinical outcomes.
• **Early rehabilitation and structured fixation strategies** enhance joint stability and functional recovery over time.
• **Long-term follow-up (24 months)** demonstrates significant improvement in MEPS, DASH, and OES scores, with near-complete restoration of elbow motion.
• **High bone consolidation rates** were observed, with no cases of implant failure or major complications.
• **Future prospective studies** with larger sample sizes are needed to further validate this surgical protocol and optimize treatment strategies.

## 1 Introduction

Transolecranon fracture-dislocations are rare and complex elbow injuries characterized by a fracture through the olecranon with associated coronoid and radial head fractures, often combined with posterior elbow dislocation. This injury pattern disrupts all major osseous stabilizers of the elbow while sparing the proximal radioulnar joint, differentiating it from Monteggia fractures. Its uniqueness lies in the combination of high-energy trauma, anatomical complexity, and biomechanical instability, which make surgical treatment particularly challenging. Despite its severity, this condition remains underreported, with limited standardized approaches available in the literature. Our study addresses this gap by analyzing outcomes following a reproducible surgical protocol in a series of 12 cases.

**Fig. 1:**
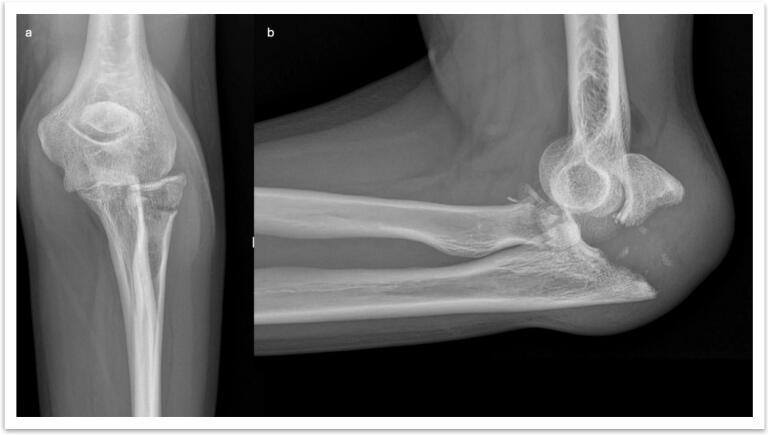
Terrible tetrad of the elbow; a) AP X-ray of the left elbow; b) Lateral X-ray of the right elbow showing the terrible tetrad of the elbow.

**Fig. 2:**
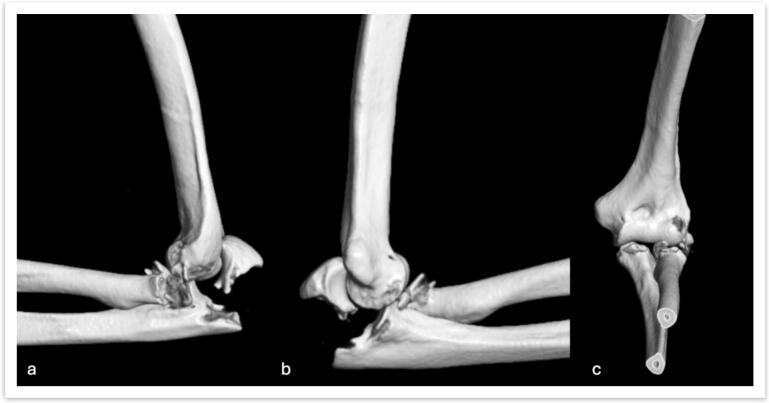
3D reconstruction of the terrible tetrad of the elbow injury, in its projections: a) Lateral, b) Medial, and c) Anteroposterior.

Elbow fracture-dislocations are complex injuries that challenge orthopedic surgeons due to their inherent instability and high risk of complications [1,2]. Among these, transolecranon fracture-dislocations represent a distinct subset, differing from Monteggia fractures and the terrible triad of the elbow [3,4]. The term “terrible tetrad of the elbow” was first described by Cavaglia et al. [5] to define a transolecranon fracture with concomitant fractures of the coronoid process and radial head, combined with a posterior elbow dislocation (Fig. 1, Fig. 2).

Unlike typical posterior elbow dislocations, where the ulna and radius translate posteriorly relative to the humerus, transolecranon fracture-dislocations occur through a different mechanism. The primary injury force is a high-energy axial load applied to the elbow in a semiflexed position, causing a fracture of the greater sigmoid notch. As the humerus translates anteriorly through the fractured olecranon, it impacts the coronoid process and radial head, leading to their respective fractures [6,7]. Despite this extensive bony disruption, the proximal radioulnar joint remains intact, differentiating these injuries from Monteggia fracture-dislocations, which involve radioulnar dissociation [8,9].

Given the severe instability of these injuries, treatment requires more than anatomical reduction alone. A systematic surgical approach is essential to optimize outcomes, restore joint congruency, and facilitate early rehabilitation. This study aims to analyze the clinical and functional outcomes of 12 cases of transolecranon fracture-dislocations treated with a standardized surgical protocol, emphasizing the importance of structured management to improve reproducibility and long-term results.

## 2 Methods

### 2.1 Study design and ethical considerations

This study has been reported in accordance with the PROCESS (Preferred Reporting of Case Series in Surgery) guidelines, ensuring transparency, methodological rigor, and reproducibility in the reporting of surgical case series [10]. A retrospective case series followed the Declaration of Helsinki and did not require ethical approval per institutional guidelines, as it used de-identified patient data. The dataset is securely stored and available upon formal request from Hospital Metropolitano and Hospital Voz Andes. Since this study analyzes pre-existing records without direct patient intervention, registration in public databases was not required. All patients provided written informed consent for the use of their clinical data in research, ensuring confidentiality and compliance with ethical standards (Table 1).

**Table 1:** Characteristics of the patients studied.

Case	Age	Side	Dislocation	Coronoid fracture	Olecranon fracture	Radial fracture
1	61	Right	Posterior	I	IIB	III
2	72	Left	Posterior	I	IIB	III
3	80	Left	Posterior	III	IIA	I
4	60	Left	Posterolateral	III	IIB	II
5	63	Left	Posterior	III	IIB	I
6	73	Right	Posterolateral	III	IIB	II
7	65	Right	Posterior	I	IIB	III
8	40	Left	Posterior	II	IIA	III
9	45	Left	Posterior	III	IIB	I
10	65	Right	Posterolateral	III	IIB	II
11	68	Right	Posterior	I	IIB	III
12	21	Left	Posterior	II	IIA	III

A case series design was selected due to the rarity and complexity of trans-olecranon fracture-dislocations, which limits the feasibility of conducting large-scale cohort or cross-sectional studies. Given the low incidence of this injury pattern and the need for detailed surgical documentation, a case series allows for an in-depth analysis of surgical approaches, fixation techniques, and outcomes, serving as a valuable contribution to the limited existing literature.

### 2.2 Patient selection and inclusion criteria

Patients were included if they met the following criteria:

• Diagnosis of a transolecranon fracture-dislocation with an associated fracture of the radial head and coronoid process.
• A minimum follow-up of 12 months.
• Complete clinical and radiographic records available.

Patients with Monteggia fractures, isolated radial head or coronoid fractures, open fractures with severe soft tissue loss, or previous elbow surgeries were excluded.

### 2.3 Surgical technique

All patients underwent open reduction and internal fixation (ORIF). Surgeries were performed by two experienced orthopedic trauma surgeons, one with 30 years of experience and the other with 15 years of experience, ensuring adherence to standardized surgical principles.

To minimize inter-institutional and inter-surgeon variability, both surgeons followed a standardized surgical protocol established collaboratively prior to the start of the study. Preoperative planning, intraoperative decision-making, fixation sequence, and postoperative rehabilitation were conducted using uniform criteria at both institutions. All surgical procedures adhered to the same protocol regardless of the center, and postoperative outcomes were jointly reviewed by both surgeons to ensure consistency and reduce observer bias.

**Fig. 3:**
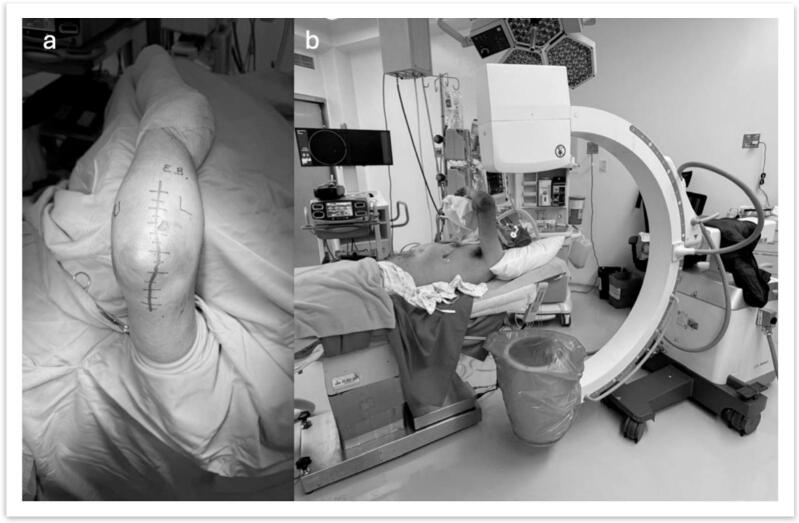
a) Wrightington posterolateral approach b) patient positioning.

**Fig. 4:**
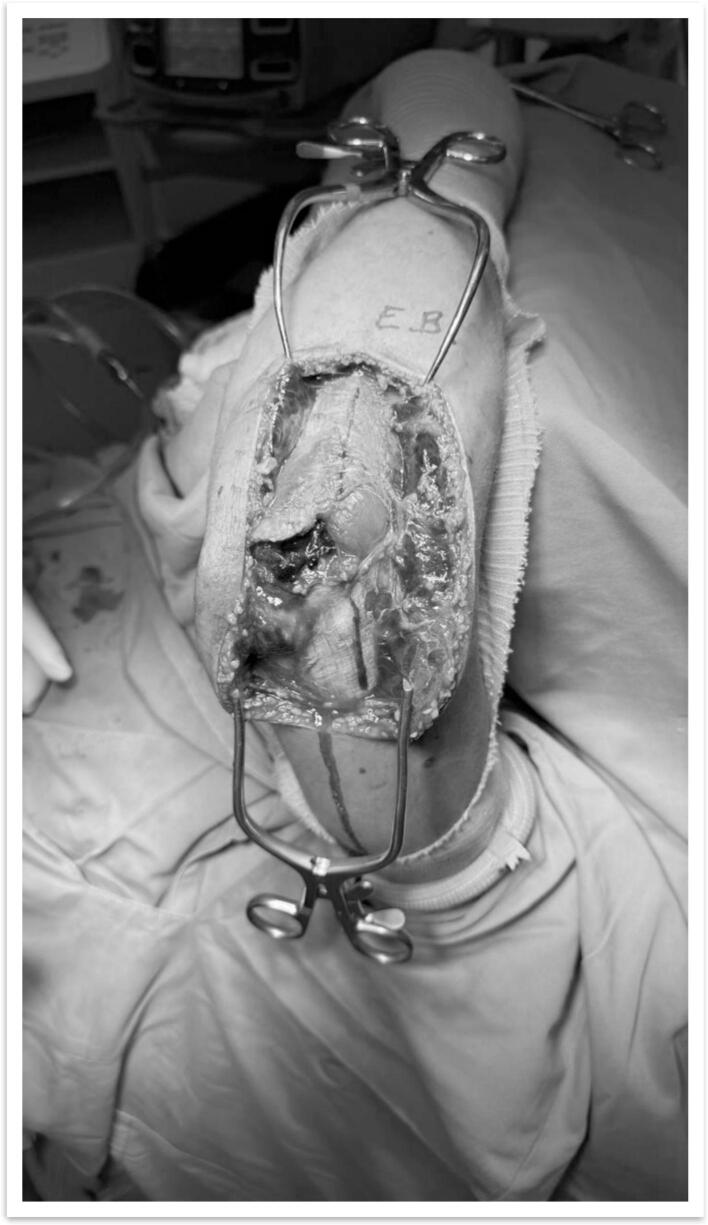
Approach used for the reduction of the terrible tetrad of the elbow, utilizing the Wrightington posterolateral approach, showing adequate exposure of the olecranon and radial head.

Surgeries were performed under general anesthesia with the patient in a supine position on a radiolucent table, the shoulder adducted, and the elbow flexed over the torso, supported by an armrest (Fig. 3, Fig. 4). Two surgical approaches were employed:

• **Wrightington Posterolateral Approach with Osteotomy**: This was the primary approach, as it allows direct access to the radial head, olecranon fractures, and, in some cases, the anterolateral facet of the coronoid process. The osteotomy provided improved exposure of the fracture fragments and facilitated anatomical reduction.
• **Anteromedial Approach (Selective Use)**: This approach was employed only when adequate visualization and reduction of the anteromedial facet of the coronoid process could not be achieved through the posterolateral approach.

### 2.4 Fixation strategy

**Fig. 5:**
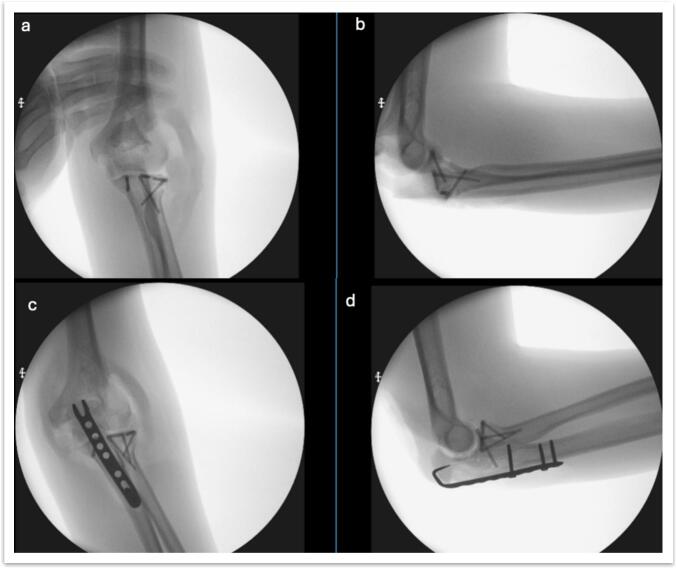
Intraoperative fluoroscopy; a and b) anteroposterior and lateral images of the fixation of radial head and coronoid process fractures; c and d) anteroposterior and lateral images of the fixation of the olecranon fracture.

**Fig. 6:**
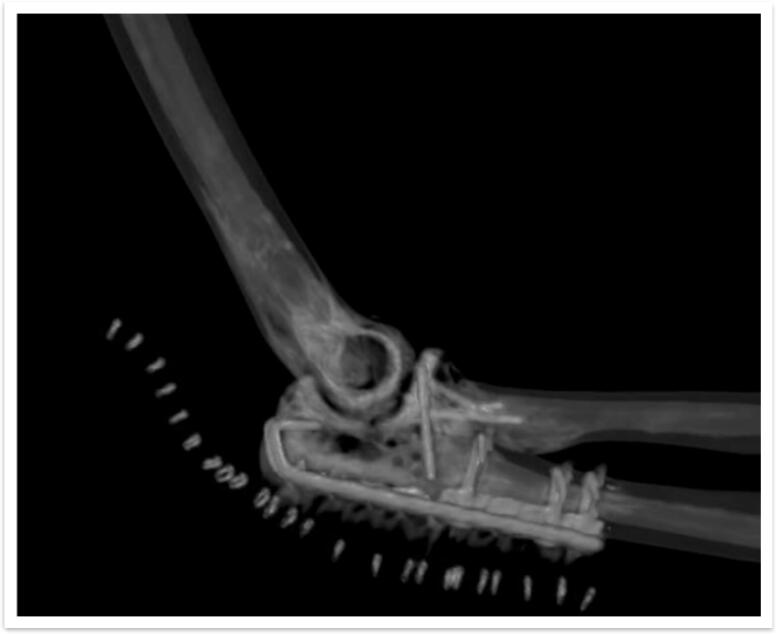
3D reconstruction of postoperative CT scan, with osteosynthesis material in red, showing adequate reduction of the articular surface of the elbow with all material in subchondral bone without affecting the biomechanics of the joint. (For interpretation of the references to colour in this figure legend, the reader is referred to the web version of this article.)

The order of fixation was standardized based on biomechanical principles: (a) fixation of the radial head and anterolateral facet of the coronoid process; (b) fixation of the olecranon fracture; (c) if necessary, an additional anteromedial approach was used for coronoid fractures, and; (d) dynamic intraoperative testing was performed to assess stability, with ligament repair when indicated (Fig. 5, Fig. 6).

Fixation was performed using rigid constructs tailored to each fracture component. For olecranon fractures, pre-contoured low-contact dynamic compression plates (LCP Olecranon Plate, Synthes®) or sharp-teeth olecranon hook plates (TriMed®) were employed to ensure stable fixation along the dorsal aspect of the proximal ulna. Implant selection was based on the degree of comminution, fracture morphology, and bone quality.

Coronoid process fractures were addressed using either 1.5 mm or 2.0 mm mini-fragment metacarpal plates (TriMed®), pre-molded to conform to the anteromedial or anterolateral facets, or with headless compression screws (Acutrak Mini®) when fragment size and accessibility permitted. Radial head fractures were similarly treated using headless compression screws or low-profile plates, selected according to the Mason classification and intraoperative reducibility. This approach aimed to restore joint congruency, anterior column stability, and trochlear notch anatomy while maintaining consistency across all cases.

### 2.5 Quality control

To ensure quality and reproducibility, a standardized surgical protocol was followed, including:

• Preoperative planning with radiographic and CT imaging to classify fracture patterns and define the optimal surgical approach.
• Standardized fixation sequence based on biomechanical principles to maximize joint stability.
• Intraoperative fluoroscopic verification of reduction quality and implant positioning.
• Postoperative imaging review by both surgeons to confirm alignment, healing progression, and detect early complications.

### 2.6 Postoperative management and rehabilitation

Postoperatively, a brachio-palmar plaster splint was applied for two weeks, with the elbow immobilized at 90° flexion and the forearm in pronation. Supervised rehabilitation was initiated in the second postoperative week. Patients received celecoxib 200 mg twice daily for three weeks to prevent heterotopic ossification and analgesia with paracetamol 1000 mg three times a day, with tramadol as needed.

### 2.7 Evaluation and follow-up

**Fig. 7:**
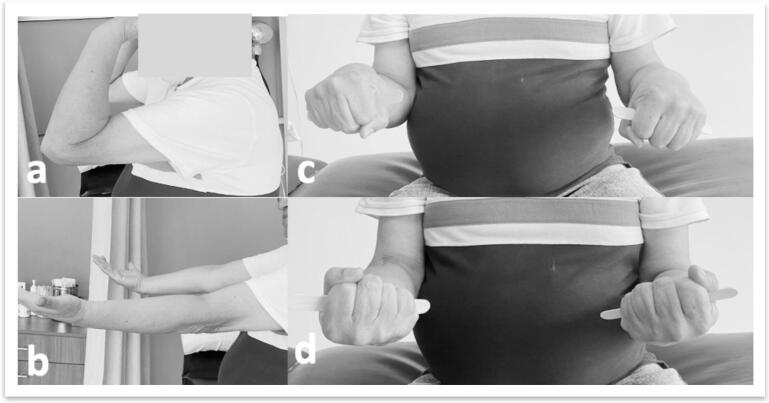
a-b) Flexion-extension range of motion at 2-year follow-up; c-d) Pronation-supination range of motion at 2-year follow-up.

Patients were followed up for a minimum of 12 months and a maximum of 24 months (Fig. 7). Clinical assessments included:

• Patient-Reported Outcome Measures (PROMS): MEPS, DASH, and OES were assessed at 2, 6, 12, and 24 months.
• Radiographic Analysis: X-rays were used to assess bone consolidation, synostosis, heterotopic ossification, joint congruence, and post-traumatic arthritis (Broberg-Morrey classification).
• Range of Motion (ROM): Flexion-extension and pronation-supination were recorded at each follow-up visit.

### 2.8 Statistical analysis

Descriptive statistics were used to summarize patient demographics, injury characteristics, and postoperative outcomes. A repeated-measures ANOVA was performed to evaluate improvements in functional scores (MEPS, DASH, and OES) over time (2, 6, 12, and 24 months). This test was chosen because it allows for the analysis of within-subject variations across multiple time points, making it appropriate for evaluating changes in functional outcomes over time within the same patient cohort.

A Greenhouse-Geisser correction was applied when sphericity assumptions were violated. Statistical significance was set at *p* < 0.05. The analysis was conducted using SciPy version X (Python), ensuring methodological rigor and reproducibility.

## 3 Results

This study analyzed 12 patients with transolecranon fracture-dislocations, assessing their clinical and functional evolution over a 24-month follow-up period. A standardized surgical protocol involving internal fixation and early rehabilitation was implemented. The mean patient age was 64.3 ± 11.9 years, ranging from 21 to 80 years, indicating that this pathology predominantly affects older adults. The distribution between affected sides was equal, with six cases involving the left side and six involving the right. Regarding dislocation type, 75 % of cases presented posterior dislocations, while 25 % were posterolateral.

Concerning the fracture pattern, type III coronoid fractures were observed in 50 % of cases, type II in 25 %, and type I in the remaining 25 %. Olecranon fractures were classified as type IIB in 66.7 % of cases and type IIA in 33.3 %. Radial head fractures were classified as type III in 50 % of cases, type II in 25 %, and type I in the remaining 25 %. For osteosynthesis, anatomical plates and hook plates were utilized for the olecranon, while 1.5 mm molded plates or Acutrak screws were used for coronoid and radial head fractures. A high rate of bone consolidation was achieved in all cases (Table 1, Table 2).

**Table 2:** Osteosynthesis material used for each fracture.

Case	Coronoid Fracture	Olecranon Fracture	Radial Fracture
1	–	Anatomical plate	1.5 mm plate (molded)
2	–	Hook plate	3 Acutrak screws
3	1.5 mm plate (molded)	Anatomical plate	2.0 mm T-plate
4	1 Acutrak screw	Hook plate	1.5 mm plate (molded)
5	1.5 mm plate (molded)	Anatomical plate	3 Acutrak screws
6	1 Acutrak screw	Hook plate	1.5 mm plate (molded)
7	–	Anatomical plate	2.0 mm T-plate
8	1 Acutrak screw	Hook plate	3 Acutrak screws
9	1.5 mm plate (molded)	Anatomical plate	3 Acutrak screws
10	1 Acutrak screw	Hook plate	1.5 mm plate (molded)
11	–	Anatomical plate	2.0 mm T-plate
12	1 Acutrak screw	Hook plate	3 Acutrak screws

In terms of functional recovery, a progressive improvement in the range of motion was recorded throughout the follow-up period. At two months postoperatively, mean flexion-extension was 100°/50°, indicating an initial restriction. At six months, a significant improvement was observed, reaching 125°/22°, while at twelve months, functional restoration was evident, with an average flexion-extension of 150°/5°. By twenty-four months, most patients achieved 160° of flexion with full extension, indicating near-complete restoration of joint mobility (Table 3).

**Table 3:** Flexion-extension over time.

	2 months	6 months	12 months	24 months
1	150°/50°	120°/20°	140°/5°	160°/0°
2	90°/40°	110°/15°	160°/0°	160/0°
3	90°/40°	115/ 25	145°/0°	150°/0°
4	95°/45°	120/20	145°/5°	150/0°
5	100°/55°	130/18	150°/5°	150°/5°
6	110°/40°	135/15	150°/5°	150°/5°
8	105°/50°	125/22	165°/0°	165°/0°
9	98°/42°	118/20	150°/5°	155°/5°
10	102°/48°	122/18	–	–
11	97°/45°	119/16	160°/0°	160°/0°
12	105°/50°	123/17	155°/0°	155/0°

**Fig. 8:**
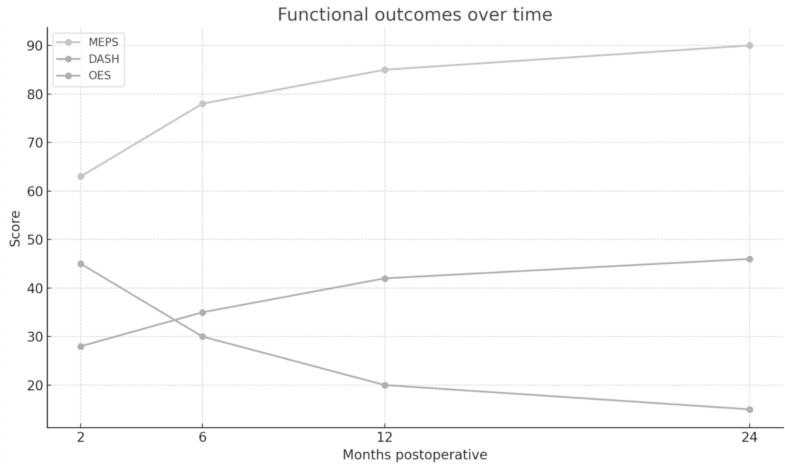
Functional outcomes over time based on MEPS, DASH, and Oxford Elbow Score (OES) at 2-, 6-, 12-, and 24-months postoperative follow-up.

Functional outcomes were assessed using three validated scoring systems: MEPS, DASH, and the Oxford Elbow Score (OES). MEPS scores progressively increased from 63 at two months to 90 at twenty-four months, reflecting excellent overall recovery. DASH scores decreased from 45 at two months to 15 at twenty-four months, indicating significant reduction in disability and optimal postoperative function. OES scores improved from 28 at two months to 46 at twenty-four months, confirming full functional restoration (Table 4, Fig. 8).

**Table 4:** PROMS over time (MEPS, DASH, OES).

	MEPS				DASH				OES			
	2 months	6 months	12 months	24 months	2 months	6 months	12 months	24 months	2 months	6 months	12 months	24 months
1	63	77	85	90	45	32	22	15	28	35	40	46
2	60	75	83	88	48	35	25	17	27	34	39	45
3	65	80	88	92	44	30	20	12	29	36	41	47
4	62	78	86	91	47	33	23	14	28	35	40	46
5	64	79	87	91	46	31	21	13	30	35	40	46
6	66	81	89	93	42	28	18	10	27	37	42	48
7	63	76	84	89	45	32	22	15	26	34	39	45
8	61	74	82	87	49	36	26	18	27	33	38	44
9	62	75	83	88	48	31	25	17	28	34	39	45
10	63	77	–	–	46	30	–	–	29	35	–	–
11	65	79	87	91	44	29	20	12	30	36	41	47
12	64	78	86	92	43	34	19	11	29	37	42	48

Mayo Elbow Performance Score (MEPS); Disabilities of the Arm, Shoulder, and Hand (DASH) score; Oxford Elbow Score (OES).

Statistical analysis, performed using repeated-measures ANOVA, did not reveal significant differences across follow-up time points (*p* > 0.05), although clinical improvement was clear throughout the evaluation period. The lack of statistical significance is likely attributable to the small sample size. These findings confirm that a standardized surgical approach, combined with a structured rehabilitation protocol, facilitates significant functional recovery and enhances elbow stability over time. The importance of precise anatomical reduction and structured fixation strategies is reinforced by these results, highlighting their role in optimizing the management of transolecranon fracture-dislocations.

## 4 Discussion

Elbow fracture-dislocations are complex injuries that pose significant challenges to orthopedic surgeons due to their inherent instability and high risk of complications [1,2]. Among these, transolecranon fracture-dislocations represent a distinct subset of injuries that differ from Monteggia fractures and the terrible triad of the elbow [3,4]. The term “terrible tetrad of the elbow” was first introduced by Caviglia et al. [5] to describe a transolecranon fracture with concomitant fractures of the coronoid process and radial head, along with a posterior elbow dislocation. As the humerus translates anteriorly through the fractured sigmoid notch during the injury mechanism, it further contributes to fractures of the coronoid and radial head [6,7].

This injury pattern results from a high-energy axial force applied to the elbow in a semiflexed position. The impact is transmitted through the olecranon, resulting in a greater sigmoid notch fracture. Subsequently, the anteriorly displaced humerus forces the trochlea against the coronoid process and the capitellum against the radial head, leading to their respective fractures [8,9]. Despite this extensive bony disruption, the proximal radioulnar joint remains intact, distinguishing transolecranon fracture-dislocations from Monteggia injuries, which involve disruption of the radioulnar articulation [11,12]. The preservation of the radioulnar joint makes transolecranon fracture-dislocations biomechanically similar to the terrible triad, as both injuries compromise the primary osseous stabilizers of the elbow, necessitating an anatomic and systematic surgical approach to restore joint congruency and function [13].

Given the inherent instability of these injuries, successful treatment requires more than just anatomical reduction. An evidence-based surgical algorithm is essential to optimize outcomes, ensure joint stability, and facilitate early rehabilitation. Recent studies have demonstrated that transolecranon fracture-dislocations can achieve functional range of movement, pain relief, and good clinical outcomes when treated with a standardized surgical protocol focused on proper ulnar fixation and restoration of dorsal angulation, in addition to addressing radial head and coronoid fractures when present [13,14].

Furthermore, Cho et al. [15], in their systematic review of transolecranon fracture-dislocations, highlighted that this injury pattern is often misdiagnosed or incorrectly classified, which can lead to suboptimal surgical decision-making. Their analysis emphasized that, unlike Monteggia fractures, transolecranon fracture-dislocations rarely involve significant ligamentous injury, reinforcing the importance of an approach centered on anatomic bony reconstruction rather than ligament repair. Additionally, they identified restoration of trochlear notch morphology as the primary surgical objective, as its disruption compromises ulnohumeral joint stability.

The significance of trochlear notch restoration was further reinforced by Mortazavi et al. [26], who retrospectively analyzed a cohort of patients with anterior transolecranon fracture-dislocations. Their study found that patients treated with reconstruction plates had superior functional outcomes compared to those treated with tension band wiring, highlighting the biomechanical advantages of rigid fixation. They reported an average Broberg and Morrey score of 89 and good-to-excellent outcomes in 87.5 % of cases, supporting the necessity of accurate anatomical restoration of the sigmoid notch as a key determinant of elbow stability. These findings align with our results, in which systematic fixation of the olecranon with rigid constructs facilitated early mobilization and optimal functional recovery.

Similarly, da Mota et al. [27] conducted a retrospective study on 25 patients with transolecranon fracture-dislocations treated with plate and screw fixation. They reported complete bone consolidation in all cases with no signs of implant failure or post-traumatic osteoarthritis. Functionally, their patients had an average flexion-extension arc of 102.6° and a pronation-supination arc of 132.8°, with MEPS and DASH scores of 89.6 and 16.5, respectively. Additionally, 84 % of patients reported no residual pain, reinforcing the importance of rigid fixation strategies and structured rehabilitation protocols to optimize postoperative recovery.

The Wrightington approach has been extensively studied and validated as an effective method for accessing the radial head. Stanley et al. [16] demonstrated that this approach provides superior visualization by preserving the annular ligament through an osteotomy of the supinator tuberosity, minimizing the risk of ligamentous damage while maintaining joint stability. Additionally, this technique allows for early mobilization, reducing postoperative stiffness.

Biomechanical studies further support the Wrightington approach over traditional posterolateral methods. Charalambous et al. [28] conducted a cadaveric study comparing the Wrightington and posterolateral approaches. Their findings demonstrated that the posterolateral approach resulted in greater valgus and varus laxity, while the Wrightington approach preserved joint stability. Additionally, following radial head excision and fixation, the posterolateral approach led to increased external rotation, whereas the Wrightington approach resulted in increased internal rotation.

In terms of fracture fixation methods, we did not restrict ourselves to a single approach but adopted a strategy tailored to the specifics of each fracture. For the fixation of coronoid process fractures, two specific techniques were used: 1.5 mm/2.0 mm metacarpal plates (TriMed®), molded as needed, and headless compression screws (Acutrak Mini®). Both methods have been supported by various studies [16,17] and compared with each other, without significant differences in clinical outcomes. However, it has been observed that the placement of Acutrak screws requires less surgical time than plate fixation.

**Fig. 9:**
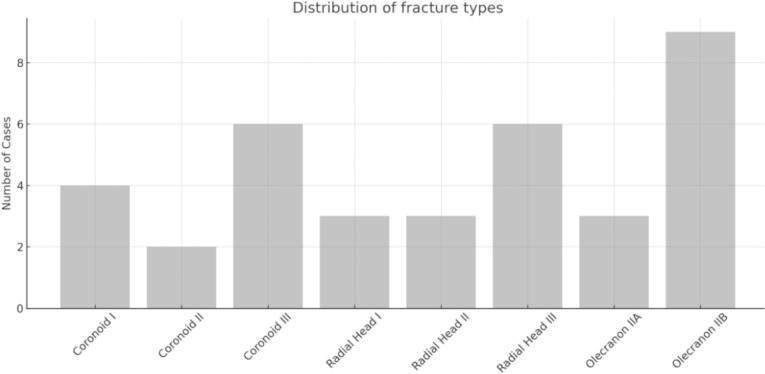
Distribution of fracture types in the cohort, classified by coronoid, radial head, and olecranon fracture subtypes.

For radial head fixation, two specific methods were employed: headless compression screws (Acutrak Mini®) and 1.5 mm/2.0 mm metacarpal plates (TriMed®), molded as necessary. A review of the literature showed that these methods have been used successfully for radial head fracture fixation [[18], [19], [20]]. For olecranon fracture fixation, two methods were used: the LCP plate for olecranon (Synthes®) and the olecranon hook plate (TriMed®). Both methods have been reported in various studies [[21], [22], [23], [24], [25]] (Fig. 9).

Statistical analysis using repeated-measures ANOVA did not reveal significant differences between the various time points (*p* > 0.05), although clinical improvement was evident throughout the follow-up period. This suggests that, while objective functional recovery was achieved, the sample size may have been insufficient to detect statistically significant differences. Similar challenges have been encountered in previous studies on transolecranon fracture-dislocations, given their low incidence and heterogeneity in fracture patterns and surgical techniques [[26], [27], [28]].

This study contributes to the surgical management of transolecranon fracture-dislocations by demonstrating that a structured approach, emphasizing sequential fixation and appropriate exposure, leads to favorable long-term outcomes. Unlike previous studies, which primarily focus on individual fixation techniques, our research provides a reproducible and systematic strategy that optimizes joint stability and facilitates early rehabilitation. Additionally, our findings support the use of a selective dual-approach technique, minimizing unnecessary surgical exposure while ensuring adequate reduction of complex fractures. By standardizing the fixation sequence, this study enhances the reproducibility of treatment and provides a framework for future comparative studies evaluating different surgical strategies.

## 5 Conclusion

Transolecranon fracture-dislocations represent a significant surgical challenge requiring a systematic and structured approach. Our findings reinforce the importance of coronoid-centered fixation strategies, optimized surgical exposure through combined approaches, and individualized implant selection. The long-term functional outcomes observed in this study suggest that this method enhances elbow stability and mobility, highlighting the need for continued refinement in the management of these complex injuries. Future research should aim to conduct multicenter prospective cohort studies with larger patient populations to validate these findings, compare surgical strategies, and refine current treatment algorithms.

## 6 Strengths

Despite the small sample size, this study has several strengths. First, it addresses a rare and underreported injury pattern with significant clinical relevance. Second, it employs a reproducible and standardized surgical protocol applied consistently across two orthopedic centers, enhancing the generalizability of the findings. Third, functional outcomes were measured using three validated PROMs at multiple postoperative intervals, providing a comprehensive evaluation of recovery. Finally, the minimum follow-up period of 12 months adds robustness to the reported outcomes.

## 7 Limitations

Despite the strengths of this study, several limitations should be acknowledged. The retrospective nature of the study inherently introduces selection bias and limits the control of confounding variables. Additionally, the small sample size (*n* = 12) reduces the statistical power, potentially explaining the lack of significance in ANOVA results despite clear clinical improvements. The variability in follow-up duration (12–24 months) may also influence long-term outcome assessments. Furthermore, the absence of a control group prevents direct comparison with alternative treatment approaches.

Additionally, further prospective studies with larger sample sizes and standardized surgical protocols across centers would help corroborate our preliminary findings and reduce confounding variables.

## Consent

Written informed consent was obtained from all patients included in this study for the publication of their clinical data. A copy of the written consent is available for review by the Editor-in-Chief of this journal upon request.

Patients' privacy was strictly maintained, and no identifying details such as names, initials, or hospital numbers were used in the manuscript.

## Ethical approval

This study was conducted as a retrospective case series and, according to institutional guidelines**,** did not require formal hospital ethics committee approval**.** Written informed consent was obtained from all patients prior to their surgical procedure, including explicit authorization for the use of their clinical and surgical data for research purposes. The study adhered to the ethical principles of the Declaration of Helsinki**, ensuring** patient confidentiality and compliance with research standards. No identifiable personal data were used in the manuscript.

Ethical approval policies of Hospital Metropolitano, Hospital Voz Andes, and their respective Institutional Review Boards were followed.

## Funding

This study did not receive any external funding. The authors confirm that no study sponsors were involved in the collection, analysis, or interpretation of data, the writing of the manuscript, or the decision to submit for publication.

## Author contribution

• **Alejandro Barros Castro, MD**: Study concept and design, data collection, data analysis, manuscript writing, surgical procedures, patient management, statistical analysis, literature review, critical manuscript revision, and final approval.
• **Edgar Barros, MD**: Study concept and design, data collection, data analysis, manuscript writing, surgical procedures, patient management, statistical analysis, literature review, critical manuscript revision, and final approval.
• **Eduardo Noboa, MD**: Data analysis, interpretation, and manuscript revision.
• **Carlos Peñaherrera, MD**: Statistical analysis, literature review, and formatting.
• **Francisco Endara, MD**: Surgical procedures, patient management, and final manuscript approval.
• **Paul Vaca Perez, MD and Diego Michilena, MD**: Critical revision of the manuscript and supervision.

## Guarantor

Alejandro Barros Castro, MD & Edgar Barros, MD accept full responsibility for the integrity of the study, had access to all data, and made the final decision to submit the manuscript for publication.

## Research registration number

This study is not a "First in Man" case report and does not require registration in a clinical trials registry.

1. Name of the registry: Not applicable
2. Unique identifying number or registration ID: Not applicable
3. Hyperlink to registration: Not applicable

## Conflict of interest statement

The authors declare no conflicts of interest related to this study. No financial or personal relationships could have influenced the work reported in this manuscript.
